# Regulation of macrophage migration in ischemic mouse hearts via an AKT2/NBA1/SPK1 pathway

**DOI:** 10.18632/oncotarget.23263

**Published:** 2017-12-15

**Authors:** Yanping Yang, Jieqiong Zhao, Juan Zhang, Yonghong Lei, Fang Yuan, Lu Liu, Haibo Gao, Hua Guo, Xiaolin Niu, Ruirui Chen, Xiaobing Fu, Yan Han, Hua Han, Tung Chan, Lianyou Zhao, Haichang Wang, Qiangsun Zheng, Xue Li

**Affiliations:** ^1^ Cardiovascular Department, Tangdu Hospital, The Fourth Military Medical University, Xian 710038, PR China; ^2^ Wound Healing and Cell Biology Laboratory, The First Affiliated Hospital, Chinese PLA General Hospital, Beijing 100853, PR China; ^3^ Department of Orthopedics, Chinese PLA General Hospital, Beijing 100853, PR China; ^4^ Department of Nutrition, Chinese PLA General Hospital, Beijing 100853, PR China; ^5^ Department of Plastic Surgery, Chinese General Hospital, Beijing 100853, PR China; ^6^ Department of Molecular Biology, The Fourth Military Medical University, Xian 710038, PR China; ^7^ Cardiovascular Department, Xibei Hospital, Xian 710038, PR China

**Keywords:** macrophage migration, atorvastatin, myocardial infarction, cardiac function

## Abstract

The role of the AKT2/NBA1/SPK1 signaling cascade in macrophage migration regulation and post-ischemic cardiac remodeling was investigated. We determined that the AKT2/NBA1/SPK1 signaling cascade regulated macrophage migration. A novel role for NBA1 in macrophage migration was discovered. Elevated AKT2 phosphorylation, NBA1, SPK1 (along with phosphorylated SPK1) levels, macrophage recruitment, apoptosis, and fibrosis were found within the infarct area. Atorvastatin had a beneficial effect on cardiac remodeling following myocardial infarction by inhibiting AKT2/NBA1/SPK1-mediated macrophage recruitment, apoptosis, and collagen deposition while increasing angiogenesis in the infarct area. Atorvastatin-related protection of cardiac remodeling following myocardial infarction was abolished in SPK1-KO mice. The AKT2/NAB1/SPK1 pathway is a novel regulating factor of macrophage migration and cardiac remodeling after myocardial infarction.

## INTRODUCTION

Improved reperfusion strategies have reduced the mortality rate following acute myocardial infarction (MI), but the prevalence of post-MI heart failure remains high. Macrophage infiltration contributes to ventricular remodeling after MI [1]. SPK1 has recently been implicated in the onset and development of post-ischemic remodeling [2]. However, the role of SPK1 in macrophage migration and cardiac remodeling is unclear. The PI3K/AKT pathway is involved in cardiac remodeling and macrophage migration [3]. The roles of SPK1, AKT, and the AKT substrate, NBA1 [4], in these processes are unclear. Pretreatment with atorvastatin was shown to reduce infarct size by an unknown mechanism [5]. We determined the effect of SPK1 and AKT/NBA1 signaling on macrophage migration and cardiac remodeling. The effects of atorvastatin on SPK1, AKT, and NBA1-mediated macrophage migration, macrophage recruitment, apoptosis, angiogenesis, fibrosis, and the mechanisms of cardiac remodeling were elucidated. Established models of myocardial infarction were employed to examine the role of AKT-NBA1 in SPK1-mediated inflammation in macrophages.

## RESULTS

### Lipopolysaccharide-induced macrophage migration upregulated AKT2 phosphorylation and NBA1 expression

**Figure 1:**
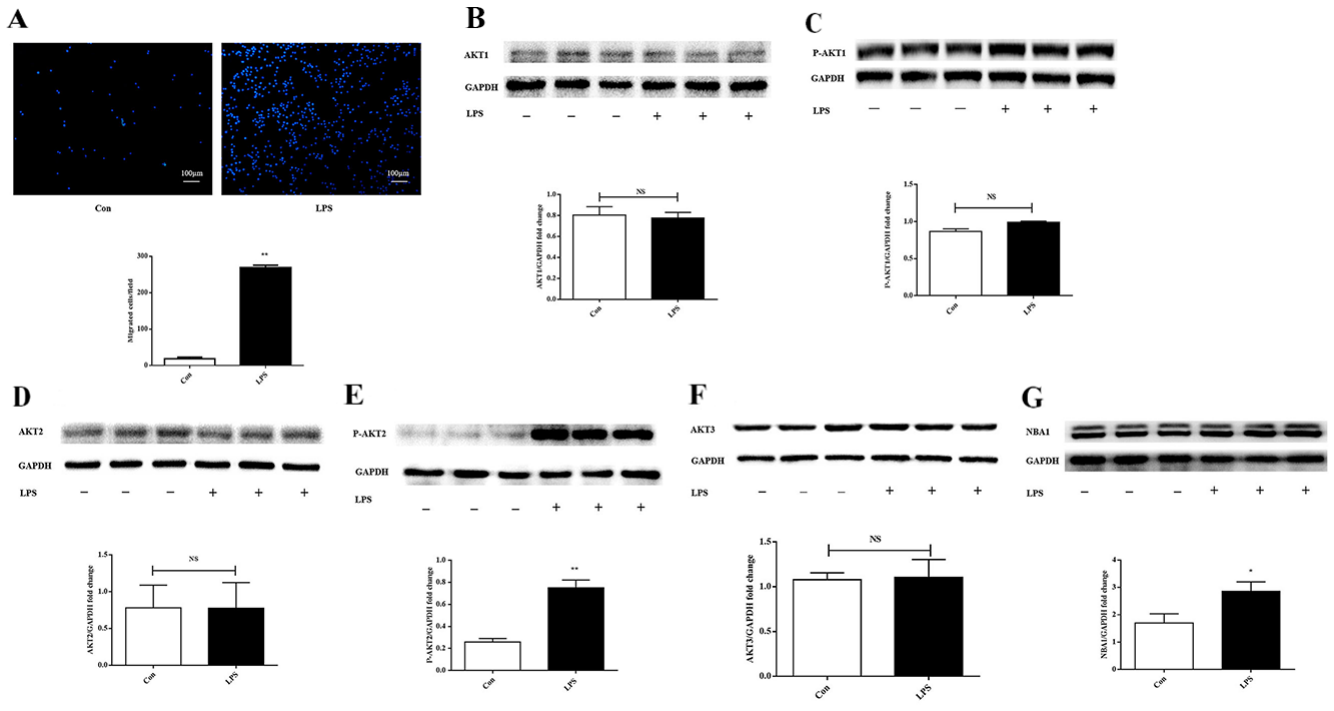
LPS increases ANA-1 cell migration, P-AKT2, and NBA1 protein expression **(A)** LPS increased migration of ANA-1 cells in a trans-well assay. Cells were grown overnight, starved for 24 h and detached. Then, 5×10^5^ cells were plated in the upper well in a serum-free RPMI 1640 medium containing 100 ng/ml LPS. After 16 h of incubation, cell migrating across the membrane were stained and counted. Four random fields were counted and the number of migrated cells was used as an index for migration. The experiment was repeated at least three times. **(B-G)** Protein expression in LPS induced ANA-1 cells. Whole cell lysates were prepared after cells were starved for 24 h and stimulated with 100 ng/ml LPS for 2 h. Immunoblots and graph shows total AKT1 (B), P-AKT1 (C), AKT2 (D), P-AKT2 (E), AKT3 (F), NBA1 (G) and GAPDH protein expression levels. Graph shows GAPDH normalized AKT1 (B), P-AKT1 (C), AKT2 (D), P-AKT2 (E), AKT3 (F), and NBA1 (G) levels. Data are presented as the mean ± SEM; n=3. ^*^P < 0.05, ^**^P < 0.01 compared with Con (Control); NS=not significant.

A transwell migration assay was performed using the murine macrophage cell line ANA-1. Lipopolysaccharide-induced macrophage migration was examined by micro-Boyden analysis (Figure 1A). AKT expression levels were evaluated in lipopolysaccharide-stimulated macrophages. Protein levels of the pan and phosphorylated AKT1, pan and phosphorylated AKT2, and pan AKT3 were evaluated by Western blot. Expression levels of AKT1, phosphorylated AKT1, AKT2, and AKT3 (Figure 1B-1F) were similar in unstimulated and lipopolysaccharide-stimulated macrophages. Elevated phosphorylation of AKT2 and upregulated NBA1 expression (Figure 1E-1G) were observed following lipopolysaccharide-induced macrophage migration.

### SPK1(P-SPK1) was upregulated in lipopolysaccharide-stimulated macrophages

**Figure 2:**
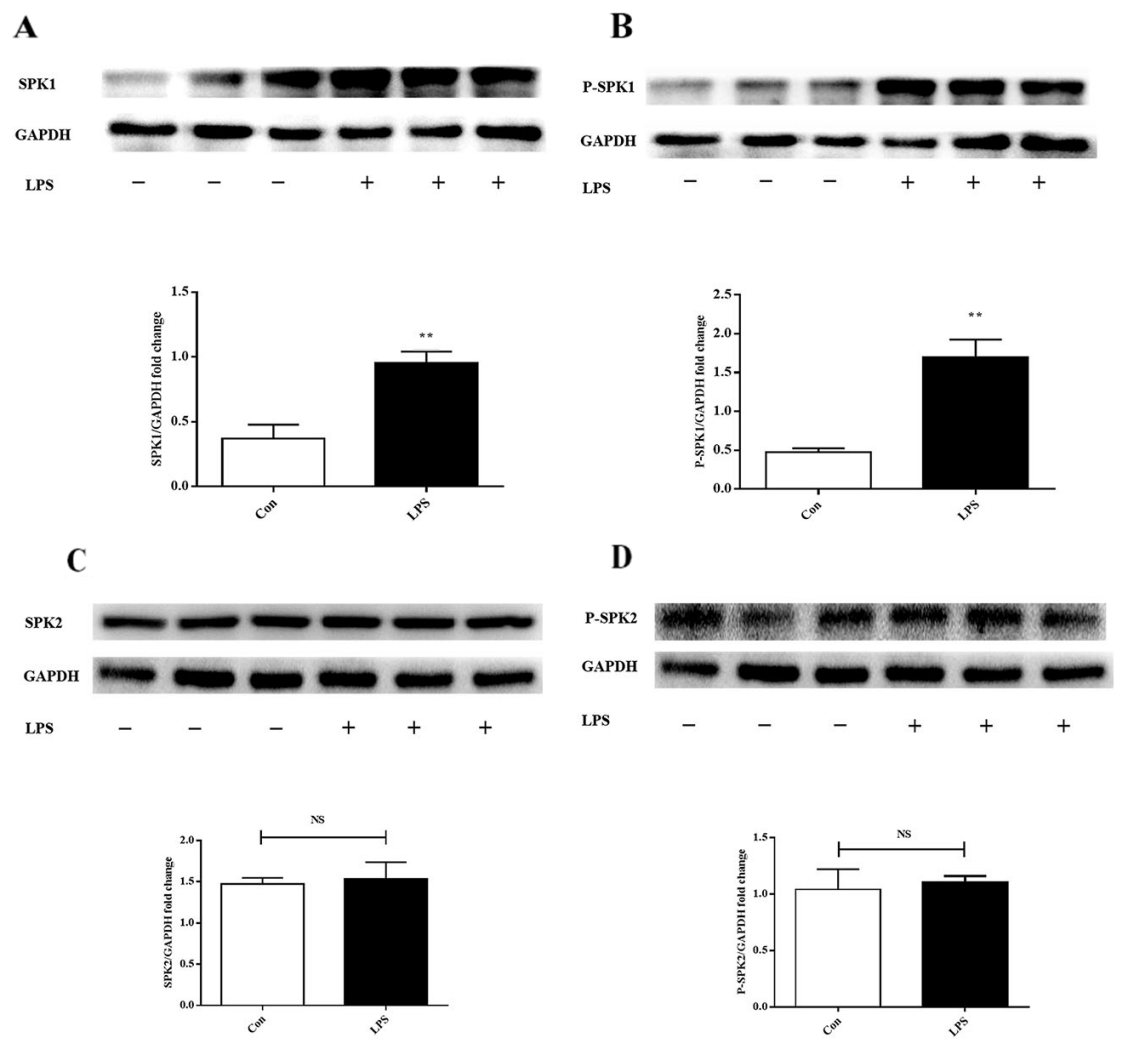
LPS increases SPK1, P-SPK1 protein expression in LPS induced ANA-1 cells ANA-1 cells were starved for 24 h and were stimulated with 100 ng/ml LPS for 2 h. Immunoblots exhibit total SPK1 **(A)**, P-SPK1 **(B)**, SPK2 **(C)**, P-SPK2 **(D)** and GAPDH protein expression levels. Graph shows GAPDH normalized SPK1 (A), P-SPK1 (B), SPK2 (C), and P-SPK2 (D) levels. Data are presented as the mean ± SEM; n=3. ^*^P < 0.05, ^**^P < 0.01 compared with Con; NS=not significant.

The effect of lipopolysaccharide on SPK1, P-SPK1, SPK2, and P-SPK2 protein expression was examined in lipopolysaccharide-challenged ANA-1 cells. Lipopolysaccharide significantly increased the levels of pan and phosphorylated SPK1 (Figure 2A-2B). SPK2 and phosphorylated SPK2 (Figure 2C-2D) expression levels were slightly varied in lipopolysaccharide-stimulated ANA-1 cells. SPK1 was crucial to lipopolysaccharide-induced macrophage migration.

### AKT2 downregulation inhibited NBA1, SPK1, and P-SPK1 expression

**Figure 3:**
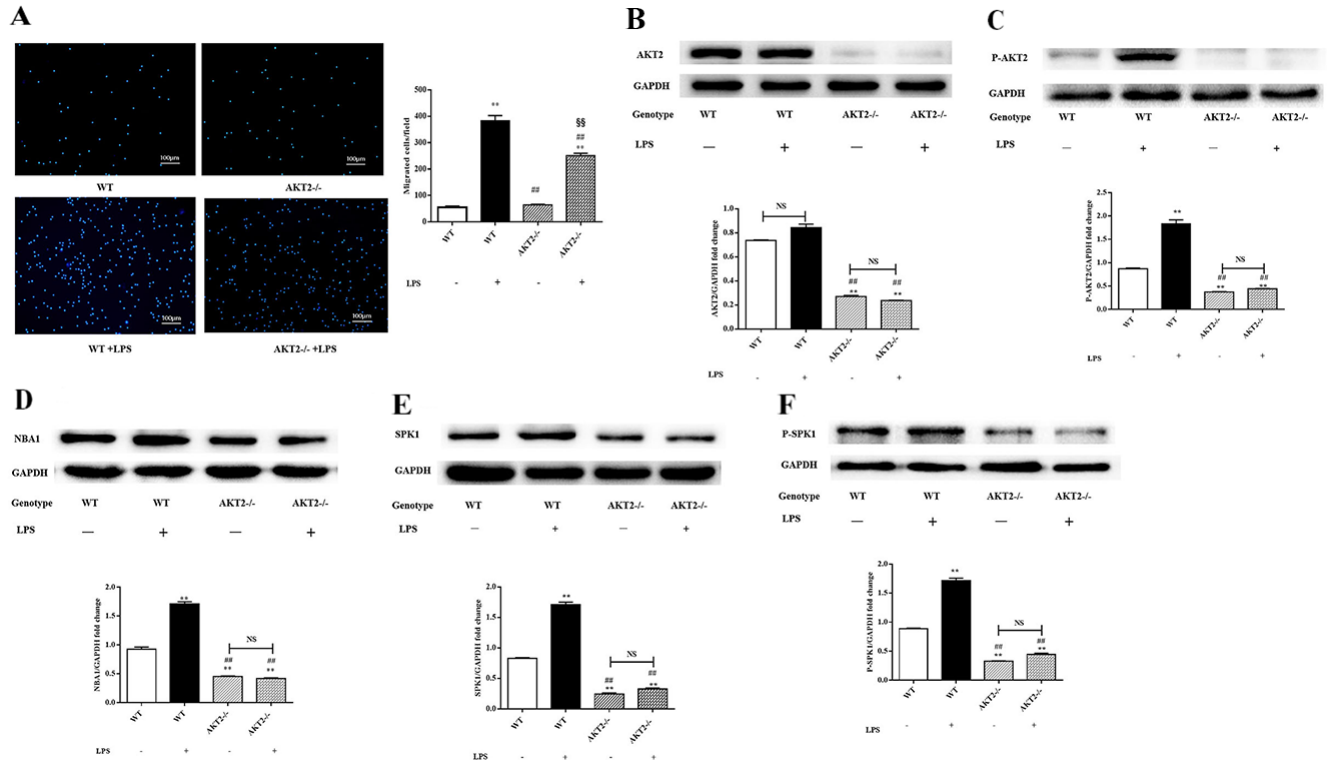
Macrophage migration, levels of AKT2, P-AKT2, NBA1, SPK1, and P-SPK1 in LPS induced murine peritoneal macrophages from WT and AKT2 KO (AKT2−/−) mice **(A)** Migration of peritoneal macrophages from WT and AKT2−/− mice. Primary cells enriched for macrophages were placed into 6-well plates (1×10^6^ cells/ml) in DMEM plus 10% FBS. After 24 h incubation and starved overnight, cells were plated in the upper well and serum-free RPMI 1640 medium containing 100 ng/ml LPS was added to the bottom well. After 16 h of incubation, cells migrating across the membrane were stained and counted. Four random fields were counted and the number of migrated cells was used as an index for migration. **(B-F)** Protein expression in LPS induced murine peritoneal macrophages. Whole cell lysates were prepared after cells were stimulated with LPS. Immunoblots show total AKT2 (B), P-AKT2 (C), NBA1 (D), SPK1 (E), P-SPK1 (F), and GAPDH protein expression levels. Graph shows GAPDH normalized AKT2 (B), P-AKT2 (C), NBA1 (D), SPK1 (E), and P-SPK1 (F) levels. Data are presented as the mean ± SEM; n=3. ^*^P < 0.05, ^**^P < 0.01 compared with WT without LPS; ^#^P < 0.05, ^##^P < 0.01 compared with WT+LPS induced group; ^§^P<0.05, ^§§^P<0.01 vs SPK1−/− without ATV treatment group; NS=not significant.

The association between AKT2 phosphorylation and NBA1 expression was examined in lipopolysaccharide-challenged peritoneal macrophages isolated from AKT2 KO (AKT2−/−) mice [6]. Limited migration was observed in lipopolysaccharide-stimulated peritoneal macrophages isolated from AKT2−/− mice (Figure 3A). AKT2, P-AKT2, NBA1, SPK1, and P-SPK1 (Figure 3B-3F) levels were decreased in peritoneal macrophages isolated from AKT2−/− mice with or without lipopolysaccharide stimulation. AKT2 phosphorylation might be an upstream signal for NBA1, SPK1, and P-SPK1.

### NBA1 downregulation decreased protein expression of SPK1(P-SPK1)

**Figure 4:**
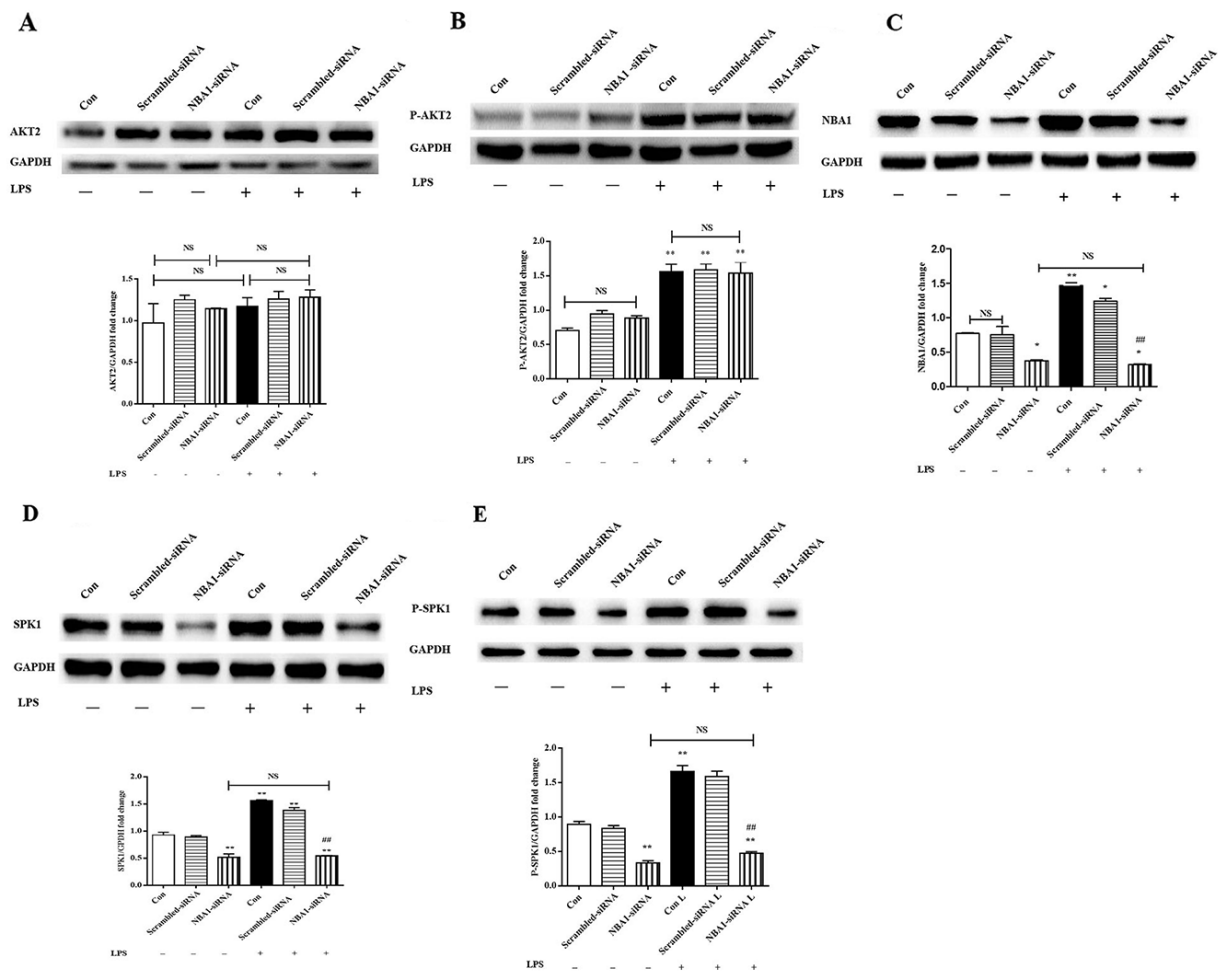
NBA1-siRNA decreased NBA1, SPK1, and P-SPK1 protein expressions, but no effect on AKT2 and P-AKT2 protein expression First, 5×10^4^ ANA-1 cells were infected by NBA1-siRNA infection solution (5×10^8^ TU) for 72h, starved overnight and then stimulated with 100 ng/ml LPS for 2h. Whole cell lysates were prepared after cells were stimulated with LPS. Immunoblots show total AKT2 **(A)**, P-AKT2 **(B)**, NBA1 **(C)**, SPK1 **(D)**, P-SPK1 **(E)** and GAPDH protein expression levels. Graph shows GAPDH normalized AKT2 (A), P-AKT2 (B), NBA1 (C), SPK1 (D), and P-SPK1 (E) levels. Data are presented as the mean ± SEM; n=3. ^*^P<0.05, ^**^P < 0.01 compared with Con without LPS; ^#^P<0.05, ^##^P < 0.01 compared Con+LPS group; NS=not significant.

Lipopolysaccharide-induced macrophages were challenged with NBA1-siRNA lentivirus infection and subsequent protein expression was assessed to examine the relationships among phosphorylated AKT2, NBA1, SPK1, and P-SPK1. NBA1-siRNA minimally effected pan and phosphorylated AKT2 (Figure 4A-4B) and decreased the levels of NBA1, SPK1, and phosphorylated SPK1 (Figure 4C-4E). AKT2 phosphorylation might be an upstream signal for NBA1. SPK1 and P-SPK1 were downstream of NBA1.

### SPK1 downregulation impaired macrophage migration

**Figure 5:**
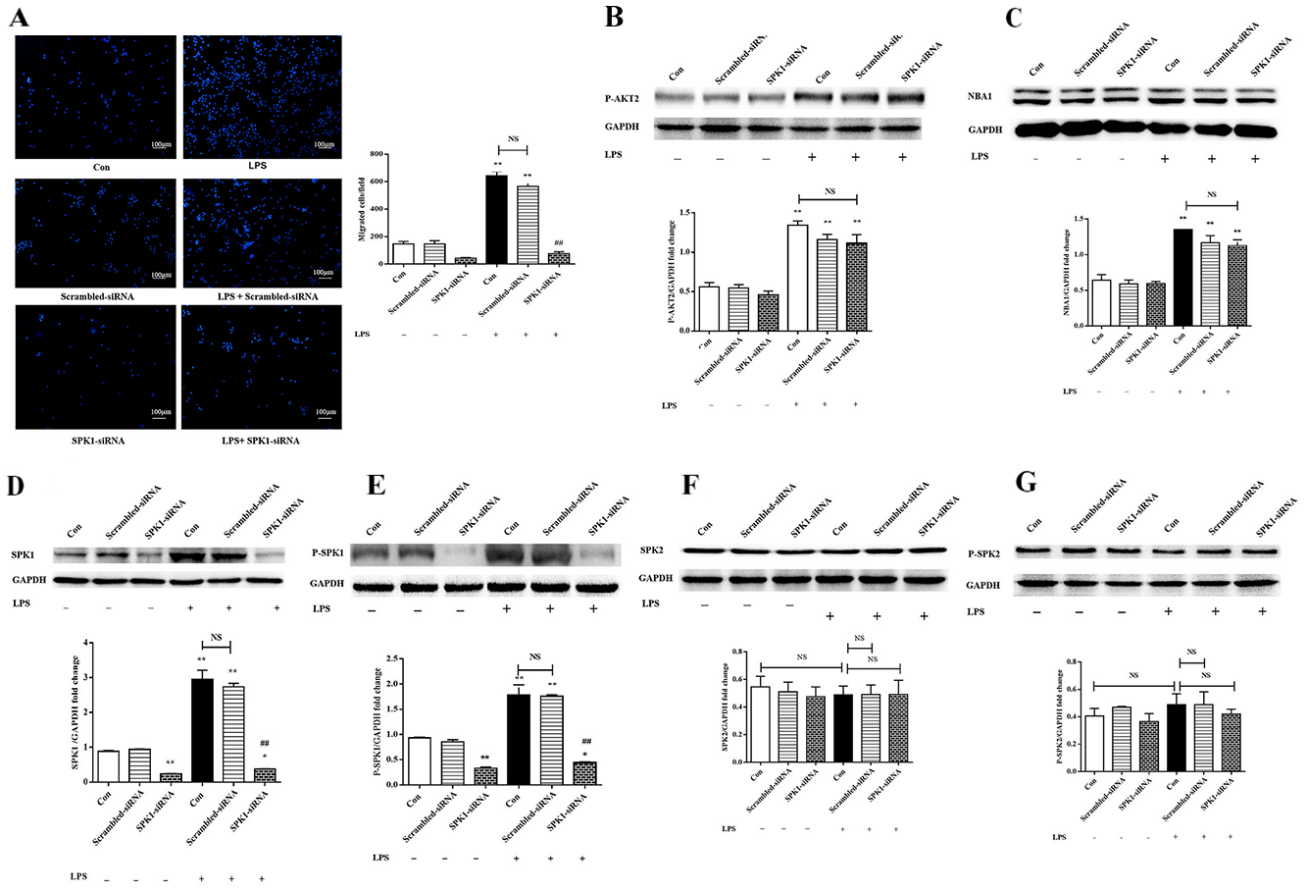
SPK1-siRNA decreased ANA-1cell migration and SPK1, P-SPK1 protein expression, but no effect on protein levels of P-AKT2, NBA1, SPK2 and P-SPK2 **(A)** SPK1-siRNA decreased migration of ANA-1 cells in a trans-well assay after LPS induction. ANA-1 cells were infected by LV-SPK1-siRNA, then cells were detached and cell suspension (5×10^5^ cells/ml) was placed in the upper well and serum-free RPMI 1640 medium with 100 ng/ml LPS was added to the bottom well. After 16 h of incubation, cells migrating across the membrane were stained and counted. The experiment was repeated at least three times with similar results. **(B-G)** Protein expression in LPS and SPK1-siRNA induced ANA-1 cells. Whole cell lysates were prepared following stimulation with LPS and SPK1-siRAN. Immunoblots showed P-AKT2 (B), NBA1 (C), SPK1 (D), P-SPK1 (E), SPK2 (F), P-SPK2 (G) and GAPDH protein expression levels. Graph shows GAPDH normalized levels of P-AKT2 (B), NBA1 (C), SPK1 (D), P-SPK1 (E), SPK2 (F), and P-SPK2 (G). Data are presented as the mean ± SEM; n=3. ^*^P < 0.05, ^**^P < 0.01 compared with Con without LPS; ^#^P < 0.05, ^##^P < 0.01 compared with Con + LPS induced group; NS=not significant.

P-AKT2, NBA1, SPK1, and P-SPK1 levels were examined following SPK1-siRNA lentiviral infection to explore the role of SPK1 in lipopolysaccharide-induced macrophage migration. SPK1-siRNA treatment decreased SPK1 protein expression and inhibited macrophage migration (Figure 5A). There was no reduction in P-AKT2 and NBA1 protein expression in lipopolysaccharide-induced macrophages treated with SPK1-siRNA (Figure 5B-5C). SPK1-siRNA reduced the protein expression levels of SPK1 and P-SPK1, but not SPK2 or P-SPK2 (Figure 5D-5G), which validated the specificity of SPK1-siRNA. SPK1 (P-SPK1) was downstream of P-AKT2 and NBA1.

### Atorvastatin suppressed macrophage migration *in vitro* via P-AKT2/NBA1/ SPK1 (P-SPK1)

**Figure 6:**
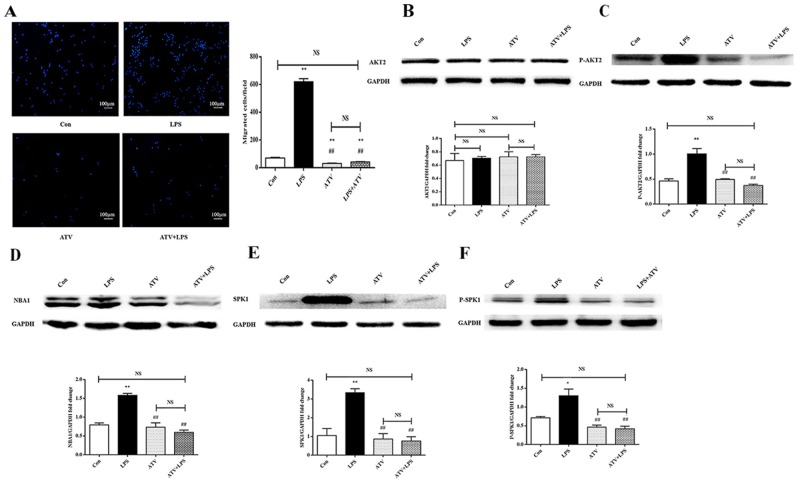
Atorvastatin (ATV) inhibits LPS-induced macrophage migration and protein expression of AKT2, P-AKT2, NBA1, SPK1, and P-SPK1 in macrophages **(A)** ATV decreases macrophage migration. ANA-1 cells were grown overnight and starved for 24 h and detached. Then, 5×10^5^ cells were plated in the upper well and serum-free RPMI 1640 medium containing 100 ng/ml LPS with or without 10 μM ATV were added to the bottom well. Cells migrating across the membrane were stained and counted. The experiment was repeated at least three times with similar results. **(B-F)** ATV decreases AKT2, P-AKT2, NBA1, SPK1, and P-SPK1 protein expressions in LPS induced macrophages. ANA-1 cells were incubated with ATV (10 μM) for 24h, then 100 ng/ml LPS induced ANA-1 cells for 2h. Then whole cell lysates were prepared. Immunoblots show AKT2 (B), P-AKT2 (C), NBA1 (D), SPK1 (E), P-SPK1 (F) and GAPDH protein expression levels. Graph shows GAPDH normalized AKT2 (B), P-AKT2 (C), NBA1 (D), SPK1 (E) and P-SPK1 (F) levels. Data are presented as the mean ± SEM; n=3. ^*^P < 0.05, ^**^P < 0.01 compared with Con group; ^#^P < 0.05, ^##^P < 0.01 compared with LPS induced group; NS=not significant.

Atorvastatin retards macrophage migration [7]. The effect of atorvastatin on AKT2, AKT2 phosphorylation, NBA1, SPK1, and SPK1 phosphorylation as well as ANA-1 cell migration was examined. ANA-1 cells were treated with atorvastatin (10 μM) for 22 h before lipopolysaccharide (100 ng/mL) stimulation for another 2 h. AKT2, P-AKT2, NBA1, SPK1, and P-SPK1 levels were examined in ANA-1 cells. Atorvastatin suppressed macrophage migration (Figure 6A) and P-AKT2, NBA1, SPK1, and P-SPK1 (Figure 6C-6F) levels without affecting AKT2 expression (Figure 6B). Atorvastatin suppressed macrophage migration by inhibiting the P-AKT2/NBA1/SPK1(P-SPK1) signaling cascade.

### Atorvastatin mediated post-MI cardioprotection via P-AKT2/NBA1/P-SPK1 inhibition

**Figure 7:**
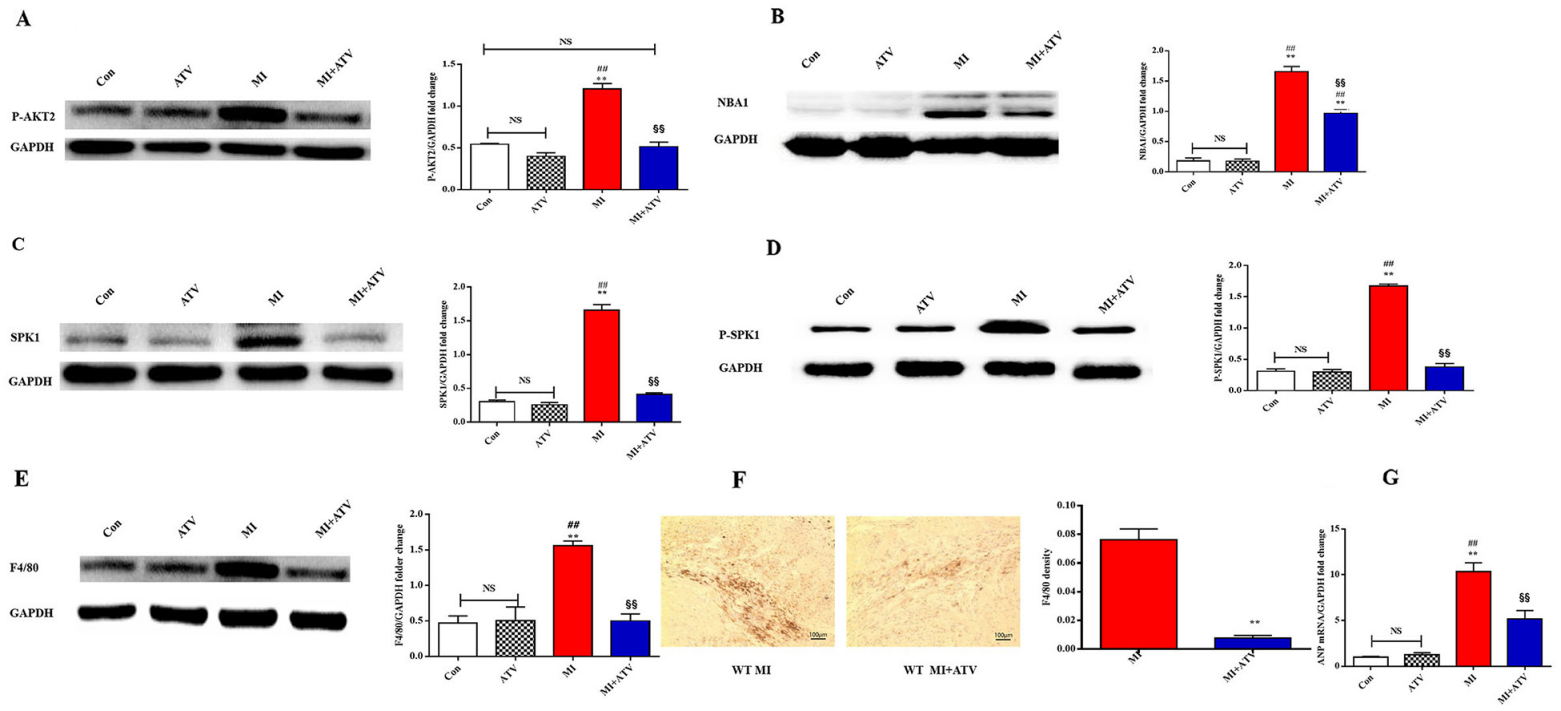
ATV ameliorated cardiac remodeling by inhibiting P-AKT2/NBA1/SPK1(P-SPK1) related macrophages recruitment in the infarction area after MI for 7 days Mice were fed ATV (10 mg/kg/day) for 1 week before and after MI-injury. We produced MI animal model. Levels of P-AKT2 **(A)**, NBA1 z**(B)**, SPK1 **(C)**, P-SPK1 **(D)**, F4/80 **(E)** protein, F4/80 density **(F)** and ANP mRNA **(G)** increased following MI injury. ATV decreased protein levels of P-AKT2 (A), NBA1 (B), SPK1 (C), P-SPK1 (D), F4/80 (E), F4/80 density (F) and ANP mRNA (G) levels in WT MI animal model. Data are presented as the mean ± SEM; n=3. ^*^P < 0.05, ^**^P < 0.01 compared with Con group; ^#^P < 0.05, ^##^P < 0.01 compared with ATV treatment group; ^§^P< 0.05, ^§§^P < 0.01 compared with MI group; NS=not significant.

We used a mouse MI model to explore the mechanism underlying the protective role of atorvastatin [8]. Atorvastatin was administered (10 mg/kg/day) to mice for 1 week before and after the MI procedure. The role of SPK1 in atorvastatin-mediated cardiac protection during remodeling was also examined. AKT2 phosphorylation, NBA1, SPK1, SPK1 phosphorylation, F4/80 protein expression, F4/80 density, and hypertrophy marker ANP mRNA expression in the infarction area were promoted at day 7 after MI without treatment and diminished by atorvastatin treatment (Figure 7A-7G).

Echocardiographic measurements showed that atorvastatin treatment increased fractional shortening and decreased LVEDD and LVESD (Table 1). Hemodynamic parameters showed that atorvastatin treatment increased +dP/dt, −dP/dt, decreased LVEDP after isoproterenol induction, and decreased HW/BW (Table 2). Atorvastatin exerted cardioprotective function by inhibiting P-AKT2/NBA1/P-SPK1-mediated regulation of macrophage recruitment in the infarction area.

**Table 1:** Mouse echocardiographic phenotype of WT vs SPK1−/−mice after MI 7 days

	Sham				MI			
	WT	WT+ATV	SPK1−/−	SPK1−/− +ATV	WT	WT+ATV	SPK1−/−	SPK1−/− +ATV
n	10	10	10	10	10	10	10	10
FS%	45.49±2.80	45.45±2.20	45.78±2.56	45.43±3.14	32.47±2.17^**^	40.15±4.15^**##^	26.76±2.81^**##††^	25.73±3.33^**##††§^
LVEDD (mm)	2.85±0.51	2.86±0.37	2.68±0.35	2.95±0.41	3.88±0.71^**^	3.67±0.26^**##^	4.21±0.15^**##††^	4.15±0.27^**##††§^
LVESD (mm)	1.55±0.29	1.56±0.20	1.45±0.19	1.60±0.19	2.62±0.48^**^	2.19±0.12^**##^	3.08±0.09^**##††^	3.07±0.13^**##††§^
HeartRate (beats/min)	438.1±19.15	450.0±25.6	439.9±19.84	433.4±10.93	436.9±10.76	441.3±18.1	430.2±7.05	440.1±10.30

Data were obtained 7 days after MI. FS%, percent fractional shorting; LVEDD, LV end-diastolic dimension in mm; LVESD, LV end-systolic dimension in mm; ^*^P < 0.05; ^**^P < 0.01 vs WT Sham; ^#^P < 0.05, ^##^P < 0.01 vs WT+M; ^†^P < 0.05, ^††^P < 0.01 vs WT+ MI+ ATV; ^§^P > 0.05 vs SPK1-KO+MI.

**Table 2:** Organ weights and hemodynamics parameters of WT vs SPK1−/− mice after MI 7 days

	Sham				MI			
	WT	WT+ATV	SPK1−/−	SPK1−/− +ATV	WT	WT+ATV	SPK1−/−	SPK1−/− +ATV
N	10	10	10	10	10	10	10	10
HW/BW(mg/g)	2.52±0.07	2.50±0.07	2.46±0.09	2.45±0.05	3.89±0.06^**^	3.25±0.09^**##^	4.48±0.11^**## ††^	4.35±0.11^**##††§^
+dP/d*t*(mmHg/s, Base)	6944±238	7060±233	6664±154	7119±168	5034±110.3^**^	5133±96.62^**Δ^	5028±101.7^**Δ^	5029±119.4^**Δ^
+dP/dt(mmHg/s, ISO)	12673±347	11928±412	12348±269	12168±410	8719±262.9^**^	10354±166.7^**##^	6337±229^**## ††^	6247±94.44^**##††§^
-dP/dt(mmHg/s, Base)	−7142±209	−7195±132.2	−7108±122.8	−7325±148.7	−5208±163^**^	−6254±95.28^**##^	4378±91.77^**##††^	−4431±107.7^**##†§^
-dP/dt(mmHg/s, ISO)	−11792 ±300	−11264 ±346	−11607 ±218	−11460 ±275	−6318±99.57^**^	−7537 ±148^**##^	−4845 ±151^**##††^	−4703 ±86.6^**##††§^
LVEDP(mmHg, Base)	3.64±0.12	3.59±0.10	3.49±0.10	3.64±0.12	11.43±0.23^**^	9.22 ±0.11^**##^	13.29±0.28^**##††^	13.11±0.28^**##††§^
LVEDP(mmHg, ISO)	4.54±0.17	4.46 ±0.18	4.18 ±0.16	4.53 ±0.14	12.4 ±0.22^**^	10.52 ±0.20^**##^	±0.40^**##††^	14.85 ±0.17^**##††§^

Base and 10 ng of isoproterenol (ISO) stimulated cardiac hemodynamic function were recorded. Data were obtained from mice 7 days after MI. HW/BW: heart weight/body weight. +dP/dt and −dP/dt: maximal 1st time derivatives of left ventricular pressure rise and fall, respectively. LVEDP: LV end diastolic pressure. ^*^P < 0.05; ^**^P < 0.01 vs WT Con; ^#^P < 0.05, ^##^P < 0.01 vs WT MI; ^†^P < 0.05, ^††^P < 0.01 vs WT MI+ATV; ^§^P > 0.05 vs SPK1−/− MI+ATV.

### Cardioprotective effects of atorvastatin were abolished in SPK1-KO mice

**Figure 8:**
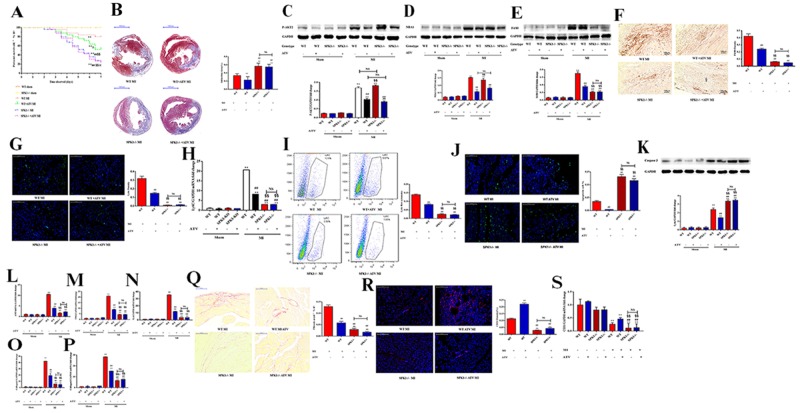
ATV protected cardiac function by reducing myocardial infarction area, macrophage recruitment, apoptosis, fibrosis, and increasing vascular density and survival curve, but ATV’s-offered protective effect on cardiac remodeling completely dampened in SPK1-KO mice WT and SPK1−/− mice were fed ATV (10 mg/kg/day) for 1 week before and after MI. Survival curve was observed between WT MI, WT + ATV MI, SPK1−/− MI, SPK1−/− + ATV MI groups. After MI 7 days, the death rate of SPK1−/− MI group is higher than that of WT MI group **(A)**. ATV treatment could decrease the death rate of WT MI model, but did not overtly lower the death rate of SPK1−/− models (A). ^*^P<0.05, ^**^P<0.01 vs WT sham without ATV group; ^#^P < 0.05, ^##^P < 0.01 compared with WT MI without ATV treatment group; ^§^P < 0.05, ^§§^P < 0.01 compared with WT MI with ATV treatment group; ^※^P>0.05 vs SPK1−/− MI without ATV treatment group. The infarction area of SPK1−/− MI group is bigger than that of WT MI group **(B)**. ATV could limit infarction area of WT MI with ATV treatment group (B). But there was no difference in the infarction area in SPK1−/− MI mice with and without ATV treatment (B). ^*^P < 0.05, ^**^P < 0.01 compared with WT MI without ATV treatment group. ^#^P< 0.05, ^##^P < 0.01 compared with WT MI with ATV treatment group. ATV could inhibit P-AKT2 **(C)** and NBA1 **(D)** protein levels in WT and SPK1−/− MI animal model. There is no difference in P-AKT2 and NBA1 protein level between WT MI and SPK1−/− MI with and without ATV treatment. ATV could inhibit F4/80 **(E)** protein levels, F4/80 density **(F)**, Ly6c density **(G)**, Ly6c mRNA **(H)**, flow cytometric analysis of Ly6c **(I)**, apoptosis density **(J)**, caspase 3 protein expression **(K)**, mRNA levels of ANP **(L)**, TNFα **(M)**, IL-6 **(N)**, collagen 1 **(O)**, collagen 3 **(P)**, collagen staining **(Q)** in the infarction area of WT MI mice. ATV could increase CD31 density **(R)** and CD 31 **(S)** mRNA level in WT MI model. But no difference in F4/80 (E) protein 6levels, F4/80 density (F), Ly6c density (G), Ly6c mRNA (H), flow cytometric analysis of Ly6c (I), apoptosis density (J), caspase 3 protein expression (K), mRNA levels of ANP (L), TNFα (M), IL-6 (N), collagen 1 (O), collagen 3 (P), collagen staining (Q), CD31 density (R), CD31(S) mRNA level were detected in SPK1−/− MI model with and without ATV treatment. Data are presented as the mean ± SEM. ^*^P < 0.05, ^**^P < 0.01 compared with WT sham without ATV sham group; ^#^P < 0.05, ^##^P < 0.01 compared with WT MI without ATV treatment group; ^§^P < 0.05, ^§§^P < 0.01 compared with WT MI with ATV treatment group; ^†^P < 0.05, ^††^P < 0.01 compared with SPK1−/− MI without ATV treatment group; NS=not significant.

We examined the role of SPK1 in cardiac remodeling in WT and SPK1−/− animal models of MI. The death rate was higher in SPK1−/− MI group than in the WT counterpart at day 7 post-MI (Figure 8A). Most SPK1−/− MI mice died from cardiac rupture. Atorvastatin treatment ameliorated cardiac remodeling and increased survival rate in the WT MI group. The death rate in the SPK1−/− MI group was not lowered with or without atorvastatin treatment (Figure 8A). Cardiac rupture and heart failure were the cause of death in these mice.

The infarction area was larger in the SPK1−/− MI group compared to the WT MI group (Figure 8B). Atorvastatin treatment limited the infarction area in the WT group, but not in the SPK1−/− MI group (Figure 8B). Atorvastatin treatment inhibited P-AKT2 and NBA1 (Figure 8C-8D) protein expression in the WT and SPK1−/− MI groups (P<0.05), but the difference between WT and SPK1−/− groups was not significant (P>0.05).

F4/80 protein expression, macrophage density, Ly6c density, Ly6c mRNA, Ly6c expression, apoptosis density, caspase 3 protein expression, ANP, TNF-α, IL-6, collagen 1, collagen 3, mRNA expression, collagen density, CD31 staining, and CD31 mRNA expression (Figure 8E-8S) were decreased in the SPK1−/− MI group and suppressed by atorvastatin treatment in the WT MI group. Apoptosis density and caspase 3 protein expression (Figure 8J-8K) increased in the SPK1−/− MI group compared with WT. However, atorvastatin treatment did not result in statistically significant differences between the SPK1−/− MI and WT groups.

Fractional shortening decreased and LVESD increased 7 days after MI in SPK1−/− mice with or without atorvastatin treatment. Echocardiographic analysis demonstrated no difference in fractional shortening, LVEDD, or LVESD in SPK1−/− mice with or without atorvastatin treatment (Table 1). Hemodynamic parameters displayed a drop in +dP/dt and −dP/dt and a rise in LVEDP after MI in the SPK1−/− groups compared to the WT groups. There was no difference in +dP/dt, −dP/dt, LVEDP and HW/BW in SPK1-KO mice with or without atorvastatin treatment (Table 2).

## DISCUSSION

AKT2 phosphorylation/NBA1/SPK1 phosphorylation was involved in macrophage migration and cardiac remodeling after MI. We demonstrated that SPK1 might serve as a beneficial cytokine during cardiac remodeling in MI animal model. Atorvastatin attenuated post-ischemic pathologic remodeling by suppressing the levels of phosphorylated AKT2, NBA1, and phosphorylated SPK1, macrophage recruitment, apoptosis, collagen deposition, and increased angiogenesis in the infarction area.

NBA1 is a primary component of the BRCA1 A complex, which contains Brca1/Bard1, Abra1, RAP80, BRCC36, and BRE. NBA1 is localized in both the nucleus and cytoplasm and helps to maintain BRE and Abra1 levels for BRCA1 recruitment to sites of DNA damage. NBA1 also contributes to multiple cell-cycle-dependent processes [9]. Our study confirmed participation of NBA1 in macrophage migration and cardiac remodeling as a potential upstream signal for SPK1(P-SPK1) and a downstream signal of AKT2 phosphorylation. These findings favor the therapeutic potential of NBA1.

A number of mechanisms have been postulated for macrophage migration including the CXCL10–CXCR3–ERK pathway [10], integrin-β1/Scr/PI3K-mediated signaling [11], PTEN-suppressed macrophage infiltration [12], AMPK/Sirt1-reduced macrophage infiltration [13], the TGF-β-activated PI3K-AKT pathway [14], and contributions from toll-like receptor signaling [1]. Sphingosine was recently implicated in the regulation of cell cycle, apoptosis, and calcium homeostasis [6]. Mammalian isoforms of sphingosine, SPK1 and SPK2, have been identified in the heart. SPK1 promoted growth and survival, whereas SPK2 suppressed proliferation by enhancing apoptosis [6]. Our data suggested that SPK1 was downstream of AKT2 and NBA1 in lipopolysaccharide-induced macrophage migration. Furthermore, knock-down of SPK1 via SPK1-siRNA inhibited macrophage migration without affecting AKT2 or NBA1 protein expression. SPK1 could be a key regulatory cytokine of macrophage migration downstream of AKT2 and NBA1.

The effects of SPK1 on cardiac remodeling have been extensively investigated. Jin and colleagues revealed a cardioprotective effect of SPK1 in ischemic postconditioning [2]. Some studies suggested that a low dose of SPK1 inhibitor *N, N*-dimethylsphingosine (DMS) was cardioprotective *in vitro* [8]. The role of SPK1 in cardiac remodeling remains controversial.

Macrophages are pivotal for wound healing with biological functions including cell debris phagocytosis, apoptosis induction, inflammatory cell and myofibroblasts recruitment, neovascularization regulation, and induction of scar formation [15]. Connective tissue formation is an essential process in the healing and repair of myocardial repair [16]. The fragile ventricular wall will undergo sudden rupture or heart failure in the absence of these connective tissues [17]. The role of macrophages in mediating the fibrotic response is complex. Excessive and prolonged infiltration of macrophages into the infarct myocardium was shown to be harmful [18]. Macrophage depletion resulted in a high mortality rate accompanied by increased left ventricular dilatation and wall thinning. Depletion of infiltrating macrophages impaired wound healing and increased mortality after myocardial injury [19].

We used a murine model of MI to explore the role of SPK1 in cardiac remodeling. Our data revealed that the survival rate of SPK1-KO MI mice was lower than WT MI mice due to the risk of cardiac rupture. The increased risk of cardiac rupture was attributed to diminished inflammation reaction, connective tissue content, and higher cell apoptosis 7 days after MI. Macrophage density in the infarction area was positively correlated with the levels of SPK1 (P-SPK1).

Atorvastatin treatment exceeded the benefits of lipid level reduction alone [20]. Numerous studies have demonstrated the beneficial role of atorvastatin in cardiac remodeling [21] and macrophage migration [7]. Our results indicated that atorvastatin might ameliorate cardiac remodeling by inhibiting myocardial infarction, macrophage recruitment, apoptosis, fibrosis, and increasing angiogenesis in the infarct area after MI injury. The protective function of statins against cardiac ischemic injury was partially attenuated in SPK1-KO mice.

**Figure 9:**
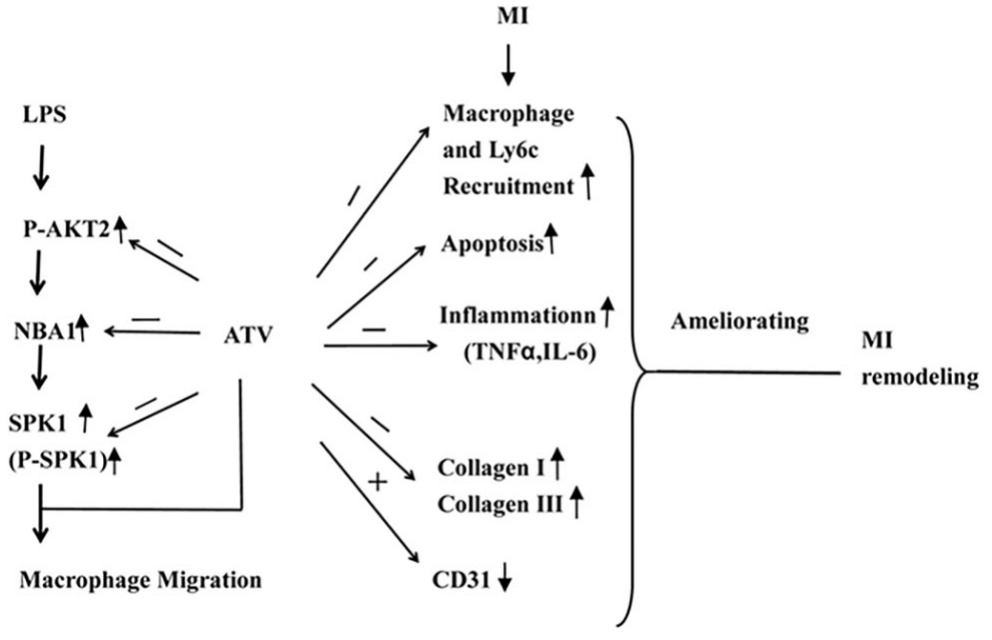
Model illustrating the mechanism of ATV-mediated MI remodeling LPS increased the protein expression levels of P-AKT2, NBA1, SPK1(P-SPK1) in LPS-induced macrophages. Myocardial infarction induced macrophages recruitment, apoptosis, inflammatory cytokine TNFα and IL-6 production in the infarction area. Moreover, ATV ameliorated myocradial remodeling by inhibiting macrophages recruitment, apoptosis, TNFα, IL-6, collagen I and III production and increasing CD31 expression in the infarction area.

Our study demonstrated that P-AKT2/NBA1/SPK1(P-SPK1) signaling regulated macrophage migration. We identified a novel role for NBA1 in macrophage migration. SPK1 was beneficial to cardiac remodeling after MI. Atorvastatin-mediated cardiac remodeling involved the inhibition of P-AKT2/NBA1/SPK1(P-SPK1)-regulated macrophage recruitment, apoptosis, fibrosis with improved angiogenesis in the infarct area. (Figure 9) These results suggest that SPK1 could be a novel therapeutic target to attenuate post-ischemic cardiac remodeling.

## MATERIALS AND METHODS

### Animal model

SPK1−/− [22] and AKT2−/− [23] mice were obtained from the Jackson Laboratory (Bar Harbor, ME, USA). SPK1−/− and AKT2−/− mice were engineered on a C57BL/6 background. Non-transgenic mouse litters served as controls, and all mice used were 8-12-week-old male mice. Mice were fed atorvastatin at 10 mg/kg/day(catalog number 134523-03-8, Sigma Aldrich, St. Louis, MO, USA) by gastric gavage for 1 week before and after MI. All animal procedures were carried out according to National Institutes of Health Guide for the Care and Use of Laboratory Animals and approved by the Animal Care and Use Committee of the Forth Military Medical University.

### Antibodies

Antibodies were directed against SPK1 (catalog number ab71700), AKT2 (ab66129), P-AKT2 (ab38513), goat anti-rabbit IgG H&L (HRP; ab6721), and GAPDH (ab9485; all from Abcam, Cambridge, MA, USA). F4/80 (catalog number sc59171), and donkey anti-goat IgG-HRP (sc2020) were from Santa Cruz Biotechnology (Santa Cruz, CA, USA). NBA1 (catalog number 12711) was from Cell Signaling Technology, Inc. (Beverly, MA, USA). P-SPK1 (catalog number AP 05255PU-N) was from DBA Acris Antibodies (Rockville, MD, USA). Caspase 3 (catalog number 19677-1-AP) was from Proteintech Group, Inc. (Chicago, USA).

### Materials

Isoproterenol was from Hospira (catalog 0409-1410-05, Hospira, UT, USA). Lipopolysaccharide (catalog number L2630) and atorvastatin calcium salt trihydrate (134523-03-8) were from Sigma Aldrich. The Ana-1 cell line was from the Cell Bank of the Chinese Academy of Sciences. Transwells were purchased from Corning Costar Company (catalog number 3422, Corning Costar, Cambridge, MA, USA).

### Isolation of murine peritoneal macrophages

Peritoneal macrophages, WT or SPK1−/−, were isolated from mice as previously described [24]. Briefly, after mice were euthanized by cervical dislocation, the body was cleaned with 75 % ethanol and a small incision was made in the abdomen. Mice were injected with 10 mL 1×PBS through the peritoneal wall into the peritoneal cavity without puncturing the intestine or any other organ. Peritoneal fluid was collected into a 15 mL tube and centrifuged at 800 g at 4°C for 5 min. The cell pellet was washed twice and resuspended in DMEM/F12 with 10% FBS, 50 μg/ml gentamicin, 50 μg/ml penicillin, and 50 ug/ml streptomycin. Cells were plated in a 60 mm petri dish and allowed to attach for 2 h in a humidified incubator with 5 % CO_2_ at 37°C. Non-adherent cells were removed by vigorously washing three times with cold PBS. Adherent cells were cultured for another 24 h for further experiments.

### SPK1 and NBA1 lentivirus and cell transfection

Production of knockdown SPK1 and NBA1 lentivirus vectors was performed as previously described [8]. GFP-encoding lentiviral strains carrying the siRNA oligonucleotides that targeted 5′-GGCAGAGATAACCTTTAAA-3′ on SPK1 mRNA and 5′-CACTCTGTCCACTGTAAAT-3′ on NBA1 mRNA were designed and cloned into a GV248 vector (Gene Chem, Shanghai, China). A scramble siRNA with the sequence of 5′-TTCTCCGAACACGTGTCACGT-3′ was cloned in parallel to create the negative control lentiviral strain. The titer of the SPK1-siRNA and the negative control was approximately 8×10^8^ TU/mL, and the NBA1-siRNA was 5×10^8^ TU/mL. ANA-1 cells were transfected by seeding 3–5×10^4^/mL ANA-1 cells (Chinese Academy of Sciences, Shanghai, China) in 6-well plates and cultured for 24 h until 30–40% were fused. Cells were starved for 24 h. Lentiviral stocks were diluted to MOI 20 with enhanced infection solution (Gene Chem, Shanghai, China) containing polybrene (5 ug/mL) according to manufacturer instructions and added to the seeded cells for 12 h with FBS-free RPMI-1640. The virus-containing medium was then replaced with fresh RPMI-1640 medium containing 10% FBS. Reporter gene expression in the lentivirus was observed 3 days after transfection via green fluorescent protein (GFP). Fluorescence microscopy (IX-53; Olympus Corporation, Tokyo, Japan) was used to detect cells that expressed GFP, and the percentage of GFP-positive cells was used to measure the transfection efficiency of the cells.

### Macrophage migration

Cell migration was performed in a 24-well transwell migration system (Corning) as previously described [25]. For cell migration assay, cells were pre-treated with atorvastatin (10 uM) for 24 h at 37°C. Cells were harvested, suspended in serum-free medium, and added to the upper transwell chambers (Corning, USA) at a density of 5×10^5^ cells per chamber. Serum-free medium with lipopolysaccharide (100 ng/mL) was added in the lower chamber. After 12 h incubation, the insert was removed, and cells on the upper surface were removed with a cotton swap. Cells on the lower surface were fixed with 4% paraformaldehyde in PBS for 30 min, permeabilized with 0.2% Triton X-100 in PBS for 5 min, and stained with DAPI. The stained cells were subsequently photographed and counted in each field using Image-Pro Plus Software. Total nuclei (DAPI staining, blue) in each field were counted by IP Lab Imagine Analysis Software (Version 3.5; Scanalytics, Fairfax, VA, USA). Results from different fields taken from the same slide were averaged and counted as one sample.

### Real-time quantitative polymerase chain reaction

Real-time quantitative polymerase chain reaction analysis determined gene expression. Briefly, reverse-transcribed cDNA from myocardial RNA was used to determine gene expression. Real-time PCR was performed with 50 μl containing 40 ng of genomic DNA, 250 nM of each primer, and 1× SYBR Green Master Mix. Each experimental group was performed in triplicate. GAPDH was used as a reference gene with the DCT method to quantify the results and perform statistical analysis as described previously [26]. DDCT values were then converted to fold changes in gene expression relative to WT samples. Mouse gene-specific primer sets were as follows: ANP sense, 5′-CGTGCCCCGACCCACGCCAGCATGG-3′, and anti-sense 5′-GCCTCCGAGGGCCAGCGAGCAGAGC-3′; GAPDH sense, 5′- AACGACCCCTTCATTGAC-3′; and anti-sense, 5′-TCCACGACATACTCAGCAC-3′; Ly6C sense, 5′- CTTCCTGCCCAGCAGTTACC-3′, and anti-sense 5′-TGGGCTACATGGGGACTTGT-3′; and CD31 sense, 5′-GCCAGCAGTATGAGGACCAG-3′, and anti-sense, 5′-GACCACTCCAATGACAACCA-3′.

### Animal MI model and atorvastatin treatment

The classical, ventilation-based method of MI in mice has been fully described [26]. Experiments were performed according to National Institutes of Health Guidelines on the Use of Laboratory Animals, and all procedures were approved by the Forth Military Medical University Committee on Animal Care. Mice were randomly assigned to eight groups: WT control, WT atorvastatin, WT MI, WT MI+atorvastatin, SPK1−/− control, SPK1−/− atorvastatin, SPK1−/− MI, and SPK1−/− MI+atorvastatin groups. Mice were fed atorvastatin (10 mg/kg/day) for 7 days before the MI procedure. Mice were treated with atorvastatin for 7 days after MI. Briefly, mice were anesthetized with 3% isoflurane inhalation, intubated with a 20G intravenous catheter, and ventilated with a mixture of O_2_ and 1.5–2% isoflurane using a rodent ventilator (Harvard Mini Vent 845). The stroke volume was 0.2 mL, and the respiratory rate was 120 breaths/min. Animals were placed in a supine position. A left thoracotomy was then performed through the 4^th^ intercostal space by transverse cutting of the pectoralis muscles to expose the thoracic cage. The thymus was retracted upward, and the left lung was partially collapsed. After the pericardium was opened, the left main descending coronary artery (LCA) was located and ligated with a 6-0 silk suture 2–3 mm from the origin. The ligation was confirmed successfully when the anterior wall of the left ventricle turned pale. The lungs were then inflated to displace air, and the thoracotomy site closed in layers. After 2–5 min ventilation with room air, the animal was gradually weaned from the respirator once spontaneous respiration resumed and remained in a supervised setting until fully conscious. The sham-treated animals underwent the same surgical procedures except the LCA was not occluded.

### Echocardiography

We assessed *in vivo* cardiac function at 4 weeks after MI with an echocardiographic imaging system (Vevo 770, VisualSonic, Toronto, Canada). Mice were anesthetized with 1.5% isoflurane, and two-dimensional echocardiographic views of the mid-ventricular short axis were obtained at the level of the papillary muscle tips below the mitral valve. Left ventricular (LV) internal dimensions were measured and the LV fractional shortening (LVFS) was calculated as previously described [27].

### Hemodynamic analysis of cardiac function

A 1.4 French micro-manometer-tipped catheter (SPR-671; Millar Instruments Inc.) was inserted into the right carotid artery and advanced into the LV of mice that were lightly anesthetized (i.e., maintained spontaneous respirations) with tribromoethanol/amylene hydrate (2.5% w/v, 8 μL/g, injected intraperitoneally; Avertin) For *in vivo* hemodynamic measurements. Hemodynamic parameters, including heart rate, LV end-diastolic pressure, +dP/dt, and −dP/dt were recorded in closed-chest mode at baseline and in response to 10 ng isoproterenol administered via cannulation of the right internal jugular vein [28].

### Immunoblotting

Immunoblots were performed as previously described [29]. LV tissue was homogenized in 10 volumes of lysis buffer (50 mM Tris-HCl, pH 7.4), 150 mM NaCl, 1 mM EDTA, 0.25% sodium deoxycholate, and 1% NP-40 with a protease inhibitor cocktail and phosphatase inhibitor cocktail. The homogenates were centrifuged at 15,000×g for 15 min to obtain the NP-40-soluble supernatant and insoluble pellet. Equal amounts of proteins were subjected to SDS-PAGE and subsequently transferred to nitrocellulose membranes. Membranes were scanned with the Odyssey Infrared Imaging System (LI-COR).

### Masson’s trichrome staining and infarct area calculation

Infarct area was examined by staining heart sections with the standard Masson’s trichrome method as previously described [28]. Briefly, the mice hearts were excised post-anesthesia and rinsed with phosphate-buffered saline. Heart specimens were fixed for 1–3 days in 4% paraformaldehyde at 4°C and embedded in paraffin. Serial sections of 5 μm were cut and placed on polylysine-coated glass slides. Tissue sections were deparaffinized and stained with Masson’s trichrome reagent (Sigma-Aldrich) according to manufacturer instructions. Digital photographs were taken and quantified by color threshold measures using Image J software (NIH, Bethesda, MD, USA). The infarct area was measured as the ratio (%) of the infarct area divided by the entire LV area.

### Immunohistochemical staining

Tissue sections (4–5 μm) were deparaffinized, dehydrated using a graded series of ethanol solutions, and stained with F4/80, CD31, and Ly6c. Endogenous peroxidase was inactivated with 3% hydrogen peroxide at room temperature for 20 min. Slides were soaked in 0.1 mol/L citrate buffer (pH 6.0) and placed in an autoclave at 121°C for 2 min to retrieve antigens. After washing with PBS (pH 7.4), the sections were blocked with 1% BSA diluted in PBS at 37°C for 30 min and incubated with anti-F4/80 (1:50, Abcam, Cambridge, UK) at 4°C overnight. Sections were then rinsed with PBS, incubated with HRP-conjugated goat anti-mouse antibody and DAB (DAKO, Glostrup, Denmark), washed with distilled water, incubated with 0.5% PAS for 10 min in a dark chamber, and washed with distilled water for 3 min. All sections were counterstained with hematoxylin. All immunohistochemically stained sections were observed and photographed with an Olympus microscope (IX-70 OLYMPUS, Japan). Four representative fields within each section were randomly chosen and captured under 200X magnification. The integrated optical density (IOD) in each image was measured with the same setting for all slides, and the density was calculated as IOD/total area of each image.

### Fluorescence immunostaining

Fluorescence immunostaining was performed with a sequential fluorescent method as described [30]. Primary antibodies against CD31 (1:50, Abcam, Cambridge, UK) and anti-Ly6c protein IgG (1:100, Abcam, Cambridge, UK) were used with Alexa488-conjugated goat anti-mouse IgG and Alexa568-conjugated Rabbit anti-mouse IgG (Invitrogen) as secondary antibodies. Immunofluorescence was observed with the Olympus microscope.

### Tunel assay

After myocardial infarction, the hearts were fixed in 4% paraformaldehyde in PBS for 24 h at room temperature. Fixed tissues were embedded in a paraffin block, and 4–5 μm slices were cut from each tissue block. Immunohistochemical detection of apoptotic cardiomyocytes was performed with an apoptosis detection kit (Boehringer Mannheim, Ridgefield, CT, U.S.A.) according to the manufacturer’s instructions. Myocardial apoptosis was qualitatively analyzed by detection of DNA fragmentation (DNA ladders) and quantitatively analyzed by a terminal dUTP nick end-labeling (TUNEL) assay as described previously [31]. Assays were performed in a blinded manner.

### Isolation of single heart cells

Single cells were isolated from mouse hearts according to previous methods [32]. Mice were sacrificed, mouse hearts were excised in whole, and placed in heparinized saline. The heart was finely minced into 1–2 mm pieces after removal of epicardial fatty tissue and the aorta. Blood was removed by repeated washing in heparinized saline. The tissue was digested with collagenase (1 mg/mL, Thermo Fisher Scientific), trypsin (0.1%, Gibco), and DNase I (10 μg/mL, Roche) in 10 mL RPMI media (Hyclone) for 1 h at 37°C with occasional shaking. Released cells were separated from the remaining tissue by filtration through a 100 μm nylon cell strainer (BD Falcon), washed with R10 media (RPMI-1640 supplemented with 10% heat-inactivated FBS, 2 mM L-glutamine, 25 μM 2-mercaptoethanol, 1% penicillin/streptomycin), and placed on ice. These steps were repeated twice to digest the remaining tissue. Any residual solid tissue was treated with EDTA (2 mM) in digestion media for 10 min at 37°C, collagenase (2 mg/mL) in R10 media for 30 min at 37°C, and released cells were filtered. Collected cells were pooled and pelleted at 300 g for 5 min at 4°C. Single cell suspensions were cleaned in PBS and centrifuged at 500 g for 5 min at 4°C. Cell viability (70–90%) was confirmed by the trypan blue exclusion method.

### Flow cytometry

Cell flow cytometry was performed as previously described [33]. Cells were stained with fluorophore-conjugated antibodies for 30 min on ice and washed. Cells were fixed and permeabilized after surface staining for intracellular staining using cytofix/cytoperm buffers according to manufacturer’s instructions (BD Biosciences) and stained with anti-Ly6C-FITC (BD Biosciences 553104) for 30 min at 4°C. Cells were pelleted and resuspended in staining buffer. Flow cytometry was performed with an FACSAria™ II flow cytometer (BD Biosciences) and FlowJo software version 7.6.1 was used for analysis.

### Statistical analysis

Data were expressed as the mean ± SD from at least four independent experiments or 4 mice per group. Statistical significance was determined by one-way ANOVA with Bonferroni correction for multiple comparisons or unpaired student *t*-tests. A P-value <0.05 was considered statistically significant.

## Abbreviations

SPK1: Sphingosine kinase-1
SPK2: Sphingosine kinase-2
SPK1−/−: SPK1 knockout
AKT2−/−: AKT2 knockout
MI: Myocardial infarction
FS%: Percent fractional shorting
LVEDD: LV end-diastolic dimension in mm
LVESD: LV end-systolic dimension in mm
